# Supplementary material for: Self-reported illnesses in Thatta: Evidence from a rural and underdeveloped district in Sindh province, Pakistan
Source: PLoS One. 2025 Jan 31;20(1):e0293790. doi: 10.1371/journal.pone.0293790 (PMC11785293; doi:10.1371/journal.pone.0293790)
Supplement: Attachment — Submitted filename: PONE-D-23-33013.pdf [file pone.0293790.s001.pdf]

PLOS ONE

Self-reported illnesses in Thatta: Evidence from a rural and underdeveloped district in Sindh province, Pakistan

--Manuscript Draft--

**Manuscript Number:**	PONE-D-23-33013
**Article Type:**	Research Article
**Full Title:**	Self-reported illnesses in Thatta: Evidence from a rural and underdeveloped district in Sindh province, Pakistan
**Short Title:**	Disease epidemiology of Pakistan
**Corresponding Author:**	Ashar Muhammad Malik, Ph D, M Sc, M A, PGD P A<br>Aga Khan Univeristy<br>Karachi, PAKISTAN
**Keywords:**	Self-reported Illnesses, Disease prevalence, Multiple Morbidities, determinants of health, Health surveys, Cross sectional studies
**Abstract:**	Introduction: Profiling of Self-reported illnesses (SRI) is widely used to aid priority settings in the health sector in low- and low-middle-income countries. The common source of SRI data comes from National/sub-national level surveys. However, many surveys focus on maternal and child health, and few infectious illnesses or illnesses-specific surveys. We provide complete illness profiling including chronic, infectious, and other illnesses in District Thatta.<br>Methods: A district-level survey was conducted in Thatta in 2019. A population-representative sample of all ages (n=7776) was drawn in urban and rural areas in four Talukas in Thatta. Survey questions include SRI from the respondents, confirmed by a prescription or other relevant documents. Prevalence was estimated for major and minor illnesses. Prevalence ratios were estimated for gender, age, and urban areas with a Generalized Linear Model of the Poisson family. Factors such as age, gender, poverty, and rural inhabitation contributing to multiple morbidities were explored by Zero-Inflated Poison Distribution.<br>Findings: 36.57% of the respondents to the survey reported at least one SRI. Prevalence of communicable illnesses was 20.7%, followed by non-communicable illnesses (4.8%), Gastrointestinal disorders (4.4%), and injuries/disabilities (1.9%). Urban inhabitants were more likely to have Chronic Obstructive Pulmonary Disorders (3.34, CIs 2.26-4.92) and Diabetes (1.62, CIs 0.91-2.91). Females were most likely to have injuries (1.20, CIs 0.71-2.03), disabilities (1.59, Cis 0.91-2.79), and Musculoskeletal Disorders (1.25, CIs 0.85-1.82). Children aged < 1 year (0.80, CIs 0.63-0.96) and elderly >65 years (0.78, CIs 0.56-1.01) were more likely to have comorbidities and rural inhabitants were less likely to have comorbidities (-0.51, CIs -0.59- -0.44).<br>Discussion: The findings of this research serve as a tool for priority settings in the health sector by the district health administration. For multiple reasons, our estimates of disease burden are higher and are more comprehensive than the current evidence of health seeking in Thatta district and in Sindh province. We could not synthesize variation in over prevalence of SRIs or any specific SRI in the studies included in this review that is due to differences in survey settings or design effects. Seasonal variation of diseases was also not considered as the data was collected in the spring season.<br>Recommendation and conclusion: The burden of disease data is not routinely collected in Pakistan due to resource constraints while health surveys funded by the development partners focus on infectious diseases and maternal and child health. Our findings are timely and set an example for documenting a complete disease profile.
**Order of Authors:**	Ashar Muhammad Malik, Ph D, M Sc, M A , P G D PA
	Rahat Batool, MBBS
	Imran Naeem Abbasi, MBBS
	Zafar Ahmed Fatmi, MBBS, Ph D
	Sameen Siddiqui, MBBS, Ph D

Powered by Editorial Manager® and ProduXion Manager® from Aries Systems Corporation

	Sarah Saleem, MBBS
	Muhammad Ahmed, Ph D
**Additional Information:**	
**Question**	**Response**
**Financial Disclosure**<br><br>Enter a financial disclosure statement that describes the sources of funding for the work included in this submission. Review the submission guidelines for detailed requirements. View published research articles from *PLOS ONE* for specific examples.<br><br>This statement is required for submission and **will appear in the published article** if the submission is accepted. Please make sure it is accurate.<br><br>**Funded studies**<br>Enter a statement with the following details:<br>• Initials of the authors who received each award<br>• Grant numbers awarded to each author<br>• The full name of each funder<br>• URL of each funder website<br>• Did the sponsors or funders play any role in the study design, data collection and analysis, decision to publish, or preparation of the manuscript?<br><br>Did you receive funding for this work?	Yes
Please add funding details.<br>as follow-up to "**Financial Disclosure**<br><br>Enter a financial disclosure statement that describes the sources of funding for the work included in this submission. Review the submission guidelines for detailed requirements. View published research articles from *PLOS ONE* for specific examples.<br><br>This statement is required for submission and **will appear in the published article** if the submission is accepted. Please make	World Health Organization regional office of the Eastern Mediterranean Region supported this survey

sure it is accurate.<br><br>**Funded studies**<br>Enter a statement with the following details:<br>• Initials of the authors who received each award<br>• Grant numbers awarded to each author<br>• The full name of each funder<br>• URL of each funder website<br>• Did the sponsors or funders play any role in the study design, data collection and analysis, decision to publish, or preparation of the manuscript?<br><br>Did you receive funding for this work?"	
Please select the country of your main research funder (please select carefully as in some cases this is used in fee calculation).<br>as follow-up to "**Financial Disclosure**<br><br>Enter a financial disclosure statement that describes the sources of funding for the work included in this submission. Review the submission guidelines for detailed requirements. View published research articles from *PLOS ONE* for specific examples.<br><br>This statement is required for submission and **will appear in the published article** if the submission is accepted. Please make sure it is accurate.<br><br>**Funded studies**<br>Enter a statement with the following details:<br>• Initials of the authors who received each award<br>• Grant numbers awarded to each author<br>• The full name of each funder<br>• URL of each funder website<br>• Did the sponsors or funders play any role in the study design, data collection and analysis, decision to publish, or preparation of the manuscript?<br><br>Did you receive funding for this work?"	EGYPT - EG

**Competing Interests**<br><br>Use the instructions below to enter a competing interest statement for this submission. On behalf of all authors, disclose any competing interests that could be perceived to bias this work—acknowledging all financial support and any other relevant financial or non-financial competing interests.<br><br>This statement is required for submission and **will appear in the published article** if the submission is accepted. Please make sure it is accurate and that any funding sources listed in your Funding Information later in the submission form are also declared in your Financial Disclosure statement.<br><br>View published research articles from *PLOS ONE* for specific examples.<br><br>**NO authors have competing interests**<br><br>Enter: *The authors have declared that no competing interests exist.*<br><br>**Authors with competing interests**<br><br>Enter competing interest details beginning with this statement:<br><br>*I have read the journal's policy and the authors of this manuscript have the following competing interests: [insert competing interests here]*<br><br>* typeset	We (all authors) declared that we have no competing interests.
**Ethics Statement**<br><br>Enter an ethics statement for this submission. This statement is required if the study involved:<br><br>• Human participants<br>• Human specimens or tissue<br>• Vertebrate animals or cephalopods	Ethical Review Board of Aga Khan approved this study.

• Vertebrate embryos or tissues<br>• Field research<br><br>Write "N/A" if the submission does not require an ethics statement.<br><br>General guidance is provided below. Consult the submission guidelines for detailed instructions. **Make sure that all information entered here is included in the Methods section of the manuscript.**	

Powered by Editorial Manager® and ProduXion Manager® from Aries Systems Corporation

**Format for specific study types**<br><br>**Human Subject Research (involving human participants and/or tissue)**<br>• Give the name of the institutional review board or ethics committee that approved the study<br>• Include the approval number and/or a statement indicating approval of this research<br>• Indicate the form of consent obtained (written/oral) or the reason that consent was not obtained (e.g. the data were analyzed anonymously)<br><br>**Animal Research (involving vertebrate animals, embryos or tissues)**<br>• Provide the name of the Institutional Animal Care and Use Committee (IACUC) or other relevant ethics board that reviewed the study protocol, and indicate whether they approved this research or granted a formal waiver of ethical approval<br>• Include an approval number if one was obtained<br>• If the study involved *non-human primates*, add *additional details* about animal welfare and steps taken to ameliorate suffering<br>• If anesthesia, euthanasia, or any kind of animal sacrifice is part of the study, include briefly which substances and/or methods were applied<br><br>**Field Research**<br><br>Include the following details if this study involves the collection of plant, animal, or other materials from a natural setting:<br>• Field permit number<br>• Name of the institution or relevant body that granted permission	
**Data Availability**<br><br>Authors are required to make all data underlying the findings described fully available, without restriction, and from the time of publication. PLOS allows rare exceptions to address legal and ethical concerns. See the PLOS Data Policy and FAQ for detailed information.	No - some restrictions will apply

Powered by Editorial Manager® and ProduXion Manager® from Aries Systems Corporation

A Data Availability Statement describing where the data can be found is required at submission. Your answers to this question constitute the Data Availability Statement and **will be published in the article**, if accepted.<br><br>**Important:** Stating 'data available on request from the author' is not sufficient. If your data are only available upon request, select 'No' for the first question and explain your exceptional situation in the text box.<br><br>Do the authors confirm that all data underlying the findings described in their manuscript are fully available without restriction?	
**Describe where the data may be found in full sentences. If you are copying our sample text, replace any instances of XXX with the appropriate details.**<br><br>• If the data are **held or will be held in a public repository**, include URLs, accession numbers or DOIs. If this information will only be available after acceptance, indicate this by ticking the box below. For example: *All XXX files are available from the XXX database (accession number(s) XXX, XXX.*).<br>• If the data are all contained **within the manuscript and/or Supporting Information files**, enter the following: *All relevant data are within the manuscript and its Supporting Information files.*<br>• If neither of these applies but you are able to provide **details of access elsewhere**, with or without limitations, please do so. For example:<br><br>*Data cannot be shared publicly because of [XXX]. Data are available from the XXX Institutional Data Access / Ethics Committee (contact via XXX) for researchers who meet the criteria for access to confidential data.*<br><br>*The data underlying the results presented in the study are available from (include the name of the third party*	Data is the property of Aga Khan University and can be provided for research purposes upon request.

and contact information or URL).<br>• This text is appropriate if the data are owned by a third party and authors do not have permission to share the data.<br><br>* typeset	
Additional data availability information:	Tick here if your circumstances are not covered by the questions above and you need the journal’s help to make your data available.

Powered by Editorial Manager® and ProduXion Manager® from Aries Systems Corporation

Manuscript Click here to access/download;Manuscript;HEUS-MS.docx

Title

Self-reported illnesses in Thatta: Evidence from a rural and underdeveloped district in Sindh province, Pakistan

Authors List

1. Muhammad Ashar Malik, Assistant Professor[1]
2. Rahat Batool, Resident Year II[1]
3. Muhammad Ahmed Assistant Professor[2]
4. Imran Naeem Abbasi Assistant Professor[1]
5. Zafar Fatmi, Professor[1]
6. Sarah Saleem Professor[1]
7. Sameen Siddiqi, Professor[1]

Corresponding author

Muhammad Ashar Malik, Assistant Professor[1]

Affiliations

[1] Department of Community Health Sciences, Aga Khan University, Pakistan

[2] NED University of Engineering and Technology, Pakistan

Wordcount

3046 excluding tables and figures

Acknowledgment

We gratefully acknowledge the World Health Organization, Regional Office, Eastern Mediterranean Region for providing funding for this survey. Our acknowledgments are due to the field staff of the survey for their contributions to the survey and to the officials of the district health office, Thatta for their logistic support.

1. Introduction

Compared to clinical diagnosis, Self-Reported Illnesses (SRI) are a quick and low-cost alternative for estimating the disease burden in low- and low-middle-income countries (LMICs). In many LMICs, disease patterns using the SRI framework are commonly analysed, albeit at a small scale (Giang and Allebeck et al 2003; Rehman and Gilmour et al, 2013). In Pakistan the health sector devolved to the provinces in 2010 and the demand for devolution of the health sector to the district level is growing in popularity (Suhail and Gohar et al, 2022). However, one of the major handicaps of the district administration is their limited capacity for devolved decision-making often aggravated by limited local evidence (Ali ,2018).

A rural health program was launched in Thatta district in 2017 to prototype the collaboration of academia and the district health administration. The Department of Community Health Sciences, Aga Khan University, and the District Health Office agreed to develop a partnership to accelerate efforts to improve access and use of district health services in Thatta (Abbasi and Siddiqi et al, 2021). Inception reports of RHP highlighted limited evidence as one of the reasons for weak planning and management of health services in the district (Rural Health Programme 2018). The Rural Health program planned a survey to provide district-level estimates of the complete disease profile with a population-representative sample of district Thatta.

2. Methods

2.1 Survey Settings

Thatta is an underdeveloped district in Sindh province in Pakistan. It is situated on the coastline in Lower Sindh, Pakistan (Figure 1). In the census of 2017, the population of district Thatta was 979817, the male population was 52.1% and the population density was 100/km^2.

Over 80% of the population (approximately 0.98 million), lives in rural areas (PBS, 2019), and mainly relies on agriculture and fishery for living. On the Human Development Index, it is ranked amongst the lowest: 90th out of 111 districts at the national level and 22nd among 23 districts in Sindh (NIPS, 2018). The Health Status and health-seeking patterns of the population are not so different from the Human Development Index ranking of Thatta. For example, it was ranked amongst the highest in Sindh province in terms of under-five mortality (129 deaths per 10000 live births) and malnutrition (55%) and lowest for childhood vaccination (37% of children aged 1-2 years are fully immunized). Like the national situation, the disease profile of district Thatta is limited to maternal and child health, immunization, and unknown common illnesses. The objective of the survey was to provide a full disease profile representative of district and sub-district representative levels of Thatta district.

2.2 Survey Design and Data Collection

Sample size was drawn on assuming a design effect of 1.5, a standard deviation (PKR 15300) of demand for healthcare in rural Sindh, and margin of error of 1000, and a 10% refusal rate, the final sample size of the survey was 1060 households. Multistage cluster sampling was used with a stratification strategy. This sample was distributed through sampling proportionate to population among sub-districts/Talukas (Stratum) of Thatta namely 1) Thatta, 2) Mirpur Sakro, 3) Keti Bundar, 4) Ghorabari and 5) Kharo Chann: the latter two being managed jointly (Figure 1). Each sub-district was divided into rural and urban domains (PBS, 2014). Rural and urban classification was carried out using the definition of rural union council and urban wards by the district administration of Thatta district. The primary sampling units were obtained from Union Councils and Wards in rural and urban areas respectively. Three primary sampling units (Village in Rural areas and Mohalla/street in

Urban were selected at random in each UC/Ward. In each primary sampling unit, 8-12 households (secondary sampling units) were included in the survey.

The survey questionnaire included demographic information and self-reported illness from each member of the household. Data was collected from the female and male head of the households by the male and female members of data collection teams respectively. Data was collected on a tablet using an online data collection tool namely Epi-collect, provided freely by the London School of Hygiene and Tropical Medicine (Centre for Genomic Pathogen Surveillance). SRIs were divided across sub-classifications of diseases, Communicable Diseases, Mental Health and Non-Communicable diseases, Gastrointestinal and Liver disorders, Injuries and Disabilities, Gynaecological and Obstetrics Disorders and others/unclassified Disorders. Data for CDs was collected based on past month recall, while data on NCDs were recorded if the respondent reported it at the time of the survey. Data collection started. In January 2019 and completed by April 2019.

Data was collected by trained enumerators comprising a male and a female in each team. There were eight such teams and a data collection supervisor. The data collectors were trained on data collection methods, cultural and religious sensitivities, type and classification of diseases and use of Computer Tablet to enter data in the field. Data collection was carried out in the months of January to April 2019.

Data validation included two strategies 1) weekly field visits to aid the data enumerators and directly contacting the randomly picked respondents. In addition, at the end of the survey, a validation exercise was carried out by a faculty not involved in data collection. A random sample of the ten secondary sampling units (village and ward) was drawn and in each SSU a household was contacted by telephone to validate the data collected. The results indicated successful data collection with ignorable (<3%) missing or censored information.

This survey involved data collection from human subjects reviews was carried out and approved by the institutional Ethnical Review Committee of Aga Khan University vide letter number 2018-0615-836 on 24 November 2018.

2.3 Analysis

Demographic, socio-economic characteristics and health-seeking are reported in means and proportions. Survey sampling techniques are included in all the estimates. Age classifications of the World Health Organization were used to group the respondent by their age (WHO,2014). We classified SRI into five major categories and sixteen minor categories (Table 3).

The prevalence of illnesses was estimated as a proportion of respondents that reported an illness among all respondents of the survey. Confidence intervals of prevalence were obtained by normal approximation. Prevalence ratios were estimated to account for crucial exposure variables such as gender (except in gynecological disorders) age and least developed areas. We defined Gorabari and Keti Bandar as least developed Talukas as these were ranked lowest on socio-economic indicator among the four Talukas in Thatta District.

The prevalence ratios were estimated using Generalized Linear Models of the Poison family (Coutinho and Scazufca et al, 2008). Prevalence ratios are preferred over odd ratios to overcome the problem of overestimation and difficulties in convergence of the model (Coutinho and Scazufca et al, 2008. To analyze factors influencing the multiple SRIs (0-4) we estimated the coefficient for each covariate using a Zero-inflated Poisson Distribution by a Bayesian marginal likelihood function with Laplace- Metropolis approximation (Green, 2021). All analyses, data cleaning and imputation were carried out in STATA 15.1 while data is downloaded in Excel sheets.

3. Findings

The final sample is 1392 households (7811 individuals). Females were 48% of the sample. The adult population was 58% of the sample. Most of the adults were married (62%) and illiterate (75%). One-third (30%) of adults were employed at the time of the survey. Most of the population in Thatta district was rural (81%) except for Thatta Taluka where rural inhabitation was 67%. As opposed to the district average of literacy (23%) in Thatta taluka literacy was almost one-third of the population (30%). Among Talukas, the proportions of females and employed in Keti Bander, literate and living in urban areas in Thatta, married in Mirpure Sakro were higher than other talukas and the district average.

Nearly 37% of the respondents reported at least one SRI: 21% communicable diseases, 5% non-communicable diseases, 4% gastrointestinal and Liver diseases, 2% injuries and disabilities, and 2% other diseases, while 3% of the women of reproductive age reported pregnancy-related health care needs. Among all SRIs, Malaria/fever and flu/cough most common illnesses reported by the respondents (10 %) followed by upper respiratory tract infections.

The estimated prevalence ratios revealed that being a female (PR 1.2, CIs 1.13-1.27), aged over 60 years (PR 1.54, CIs 1.41-1.69) and under five years (PR 1.42, CIs 1.33-1.52) are more likely to report an SRI. Living in urban (PR 1.51, CIs 1.42-1.61) and from least developed areas (PR 1.37, CIs 1.29-1.45) were more likely to report an SRI (Figure 2). Generally being employed (PR 0.83, CIs 0.75-0.91) and living in large/extended families (PR 0.7, CIs 0.66-0.74) decreased the likelihood of falling ill in Thatta district. The prevalence ratios of Diabetes (PR 7.78, CIs 4.71-12.84) and Arthritis/musculoskeletal disorders (PR 4.91, CIs 2.55-9.47) in over 60 years were among the highest.

The factors that determine the multiple morbidities were similar as the factors determining being ill and types of diseases/disorders except for gender. Being a female decreases the

probability of multiple morbidities (mean -0.16, CIs -0.29- -0.04) in Thatta district. Living in an urban increased the probability of multiple morbidities by 42% (CIs 0.3-0.51) followed by living in the least developed areas (Regression Mean 0.29, CIs 0.15-0.41) whereas being currently employees and living in an extended family decreased the probability of multiple morbidities by 23% and 22% respectively.

4. Discussions

To the best of our knowledge, the most recent burden of disease survey prior to this study was carried out in Pakistan in 1994 namely National Health Survey (PMRC,1994). This study provides robust district-level representative estimates of the prevalence of SRI that are crucial for evidence-based priority setting in district Thatta. The findings in this study depict an alarming picture of health that contrasts with findings from national and provincial surveys for the self-reported prevalence of illnesses. These findings advocate the importance of local evidence for decentralized planning and management. However, there are certain limitations to the interpretation of the results. Firstly, this survey was a rapid cross-sectional survey conducted in spring and did not capture seasonal variation in the burden of disease. Secondly, findings on the prevalence of disease are based on verbal autopsy: though with validation techniques such as a record of formal consultation, enquiring for laboratory or radiographic findings, etc., no clinical examination was conducted by the enumerators during the data collection.

The small-scale rapid survey design used in this survey is similar to SRI surveys carried out in 1-3 districts Giang and Allebeck (2003), Ir and Men (2010), Rehman and Gilmour et al, 2013 and Paudel (2020). Using separate recalls for data collection, “ever diagnosed” for non-communicable/ Chronic diseases and four weeks for communicable/acute diseases is unique to this survey. As from our literature search, we find that the health surveys on SRIs used

common recall for all diseases NCDs and CDs (Giang and Allebeck 2003; Ir an Men, 2010; Rehman and Gilmour et al, 2013; and Paudel,2020) and acute/ communicable disease survey by SeoAung and MyintOo et al (2015) used a 90 days recall. Disease surveys that focused on NCDs enquired about diseases based on "ever diagnosed/ informed by a physician or health worker" (Van Minh and Ng et al, 2008; Letamo and Keetile et al, 2017; Camacho and Gomez-Arbelaez et al, 2020; Van Minh and Long et al, 2020; Siddharthan and Kalyesubula et al, 2021).With the exception of Rehman and Gilmour et al, (2013), SRIs surveys that included all diseases were carried out in rural areas (Giang and Allebeck 2003; Ir an Men, 2010; and Paudel,2020). Surveys on NCD, on the other hand, were carried out in urban areas SeoAung and MyintOo et al (2015) or were on large-scale (Van Minh and Ng et al, 2008; Letamo and Keetile et al, 2017; Camacho and Gomez-Arbelaez et al, 2020).

A common feature of SRI surveys that we included in our literature review is that these were carried out in Low-and Low-middle income countries, Colombia (Camacho and Gomez-Arbelaez et al, 2020), Vietnam (Giang and Allebeck 2003; Van Minh and Long et al, 2020), Botswana (Letamo and Keetile et al, 2017), Nepal (Paudel,2020), Bangladesh (Rehman and Gilmour et al, 2013), Myanmar (SeoAung and MyintOo et al, 2015), Uganda (SeoAung and MyintOo et al (2015). A possible explanation for this trend is that the burden of disease data is not routinely collected in many LMICs. Moreover, the health surveys are expensive and in LMICs, such surveys are funded by the development partners restricting data collection to infectious diseases and maternal and child health such as Living Standard Surveys sponsored by the World Bank, Multi-Indicator Cluster Surveys by UNICEF, and Demographic and Health Surveys by the USAID.

Estimates of the lone Burden of Disease study by the Pakistan Medical Research Council, in 1994 for NCDs (37.7%) and CD (38.4%) (PMRC, 1994) were comparable, whereas in our case prevalence of CDs (20.67%) was higher than the prevalence of all NCDs (6.68%,)

including NCDs (4.77%) and injuries and disabilities (1.91%) in Thatta. These findings could partly be due to the disease classification used in our study and partly due to the fact Thatta is the least developed district in Pakistan while the Burden of Disease study was drawn from a nationally representative sample. In a few cases, the findings in this paper are different from the findings of national and provincial surveys with district representative sampling strategy, for example, Pakistan Social and Living Standard Measurement Survey 2019-20 and Sindh Multiple Indicator Cluster Survey 2018-19 (1). Our estimates of SRI (36.6%) are higher than the Unknown SRI in Thatta district in PSLM 2019-20 (11.12%) and MICS 2018-19 (11.02%). Disability prevalence (0.7%) in our study is lower than PSLM 2019-20 (3.01%).

We could not synthesize the variation in the prevalence of SRIs in our study nor from the literature that we could review. This was due to multiple aspects such as survey settings, design of surveys, the recall period, timing, and period of data collection etc. For example, estimates of SRI in this paper (36.6%) are lower the estimates of SRI by of Rehman and Gilmour (2013) in a urban township in Bangladesh (45%, n= 1593 households), and findings of in a rural district in Vietnam (47.7%, n=48919), but higher than Ir and Men (2010) in three rural districts in Cambodia (15.05%, n=33161) and Paudel (2020) in a rural district in Nepal (24.5%, n=6580). These studies used a rapid data collection spread over 3-4 months and focused on all ages. However, the recall period in the case of Giang and Allebeck (2003), Rehman and Gilmour (2013) and Paudel (2020) was last four weeks, while in the case of Ir and Men (2010) the recall period was last one year in Colombia. Moreover, in the case of Ir and Men (20010) the SRI data was collected by trained data collectors and was verified by a public health doctor while in the case of Giang and Allebeck (2003), Rehman and Gilmour (2013) such steps of enhancing quliaty of data collection were missing. Such variation in survey design largely influences the prevalence of a disease. For example, diabetes prevalence was found to be as low as 1.1% in Uganda as high as 5.7% in Colombia, and 9.3%

(metabolic diseases) in India. While in Colombia and India, the sample size was large: 11 districts and country level, in Uganda, the study was carried out in an urban district. The period of data collection spanned over one year in Colombia and India. In Uganda and Colombia, the sample was drawn from the adult population and in India the sample was unrestricted for age.

Our findings that women are more likely to report an illness(es) and multiple morbidities are consistent with literature reporting either proportions (Camacho and Gomez-Arbelaez et al, 2020; Giang and Allebeck 2003; Ir and Men et al 2010; Paudel, 2020; SoeAung and MyintOo et al 2015; or odd ratios (Letamo and Keetile et al 2017; Patras and Bhaise 2017; Rehman and Gilmour et al 2013; Siddharthan and Kalyesubula et al 2021; Van Minh and Ng et al, 2008) except Van Minh and Long et al, (2020) that reported odds of women (OR 0.98, CIs 0.81–1.18) reporting NCDs were lower than men in the minorities in Vietnam (Van Minh and Long et al, 2020).

Our findings suggest that respondents aged 60+ years are more likely to report SRIs or NCDs are consistent with literature findings that getting older increases prevalence or odds of NCDs (Camacho and Gomez-Arbelaez et al, 2020; SoeAung and MyintOo et al 2015, Letamo and Keetile et al 2017; Rehman and Gilmour et al 2013; Van Minh and Ng et al, 2008; VanMinh and Long et al 2020).

Our findings that living in urban areas increases the prevalence of SRIs than living in rural areas are consistent with Camacho and Gomez-Arbelaez et al (2020); Letamo and Keetile et al (2017); Patras and Bhaise (2017), particularly findings of Letamo and Keetile et al (2017) findings that in urban villages the prevalence of hypertension and Asthma was higher in urban villages than in rural areas and in cities, since the urban areas in Thatta are similar to peri-urban rather than proper urban areas with basic civic amenities. On employment status

our findings are similar to VanMinh and Long et al (2020) that report higher odds of NCDs for unemployed than employed (OR 1.59, CIs 0.96–2.69) whereas on household size our findings are supported by Rehman and Gilmour et al (2013) ORs of family size 0.89 (0.82–0.87) and Paudel, (2020) reporting of decreasing proportion with larger families (<4 members 26.8, 5-8 members 26.0% and 9+ members 20.2%).

5. Recommendations and Conclusion

For multiple reasons, our estimates of disease burden are more recent and policy relevant than the current evidence on disease profiling in Thatta district. A health survey capturing a complete disease profile is better than the disease specific surveys that are common in Pakistan. A fresh national level health survey will not only provide complete disease profiling but can unearth the relative weightage of diseases crucial for priority settings in the health sector at national and sub-national levels.

We argued that district representative data can aid better priority settings within the district. Recently, the Government of Sindh has constituted Provincial Finance Commission Award that will distribute resources among districts in Sindh. The findings of this survey provide a case study to allocate resources to districts and within district using the findings of this survey.

References

Giang KB, Allebeck P. Self-reported illness and use of health services in a rural district of Vietnam: findings from an epidemiological field laboratory. Scandinavian journal of public health. 2003;31(62_suppl):52-8.

Rahman MM, Gilmour S, Saito E, Sultana P, Shibuya K. Self-reported illness and household strategies for coping with health-care payments in Bangladesh. Bulletin of the World Health Organization. 2013;91:449-58.

Suhail A, Gohar A, Steen T. Decentralization reforms in the public health sector in Pakistan. Public Sector Reforms in Pakistan: Hierarchies, Markets and Networks: Springer; 2022. p. 195-222.

Ali, S. M. (2018). Devolution of power in Pakistan. Washington, DC: United States Institute of Peace. Available online https://www.usip.org/publications/2018/03/devolution-power-pakistan

Abbasi, I. N., & Siddiqi, S. (2021). Overcoming the challenges of community-engaged emergency referrals in a rural district of Pakistan. JPMA. The Journal of the Pakistan Medical Association, 71(11), 2648–2651. https://doi.org/10.47391/JPMA.011253

Rural Health Programme (2018). Public Health Practice, Community Health Sciences MC, Pakistan.The Aga Khan University Available online https://www.aku.edu/mcpk/chs/Pages/rural-health-programme.aspx

National Institute of Population Studies - NIPS/Pakistan and ICF. 2019. Pakistan Demographic and Health Survey 2017-18. Islamabad, Pakistan, and Rockville, Maryland, USA: NIPS and ICF.

Pakistan Bureau of Statistics. (2005). Pakistan Social and Living Standard Measurement Survey. Available online https://www.pbs.gov.pk/sites/default/files/pslm/publications/pslm2004-05/pslms%202004-05.pdf

Sindh Bureau of Statistics, 2014. Multiple Indicators Cluster Survey Sindh, 2015, Government of Sindh. 2015. Available online https://sbos.sindh.gov.pk/multiple-indicator-cluster-survey-mics

World Health Organization. (2018). Global health expenditure database. 2013. World Health Organization. Available online https://apps.who.int/nha/database

Coutinho L, Scazufca M, Menezes PR. Methods for estimating prevalence ratios in cross-sectional studies. Revista de saude publica. 2008; 42:992-8.

Green JA. Too many zeros and/or highly skewed? A tutorial on modelling health behaviour as count data with Poisson and negative binomial regression. Health Psychology and Behavioral Medicine. 2021 Jan 1;9(1):436-55.

Pakistan Medical Research Council. National Health Survey of Pakistan. In Health profile of the people of Pakistan – 1990–94 Islamabad: Network publication service; 1998.

Van Minh H, Long KQ, Ha NT, Duong DTT, Trung T, Huong HT, et al. Self-reported non-communicable diseases and associated socio-demographic status among ethnic minority populations in Vietnam, 2019. Health Psychology Open. 2020;7(2):2055102920954707.

Camacho PA, Gomez-Arbelaez D, Otero J, González-Gómez S, Molina DI, Sanchez G, et al. Self-reported prevalence of chronic non-communicable diseases in relation to socioeconomic and educational factors in Colombia: a community-based study in 11 departments. Global Heart. 2020;15(1).

Letamo G, Keetile M, Navaneetham K, Phatsimo M. Prevalence and correlates of self-reported chronic non-communicable diseases in Botswana: a cross-sectional study. International Health. 2017;9(1):11-9.

Patra S, Bhise MD. Gender differentials in prevalence of self-reported non-communicable diseases (NCDs) in India: evidence from recent NSSO survey. Journal of Public Health. 2016;24:375-85.

Click here to access/download;Figure;HEUS-FG.docx

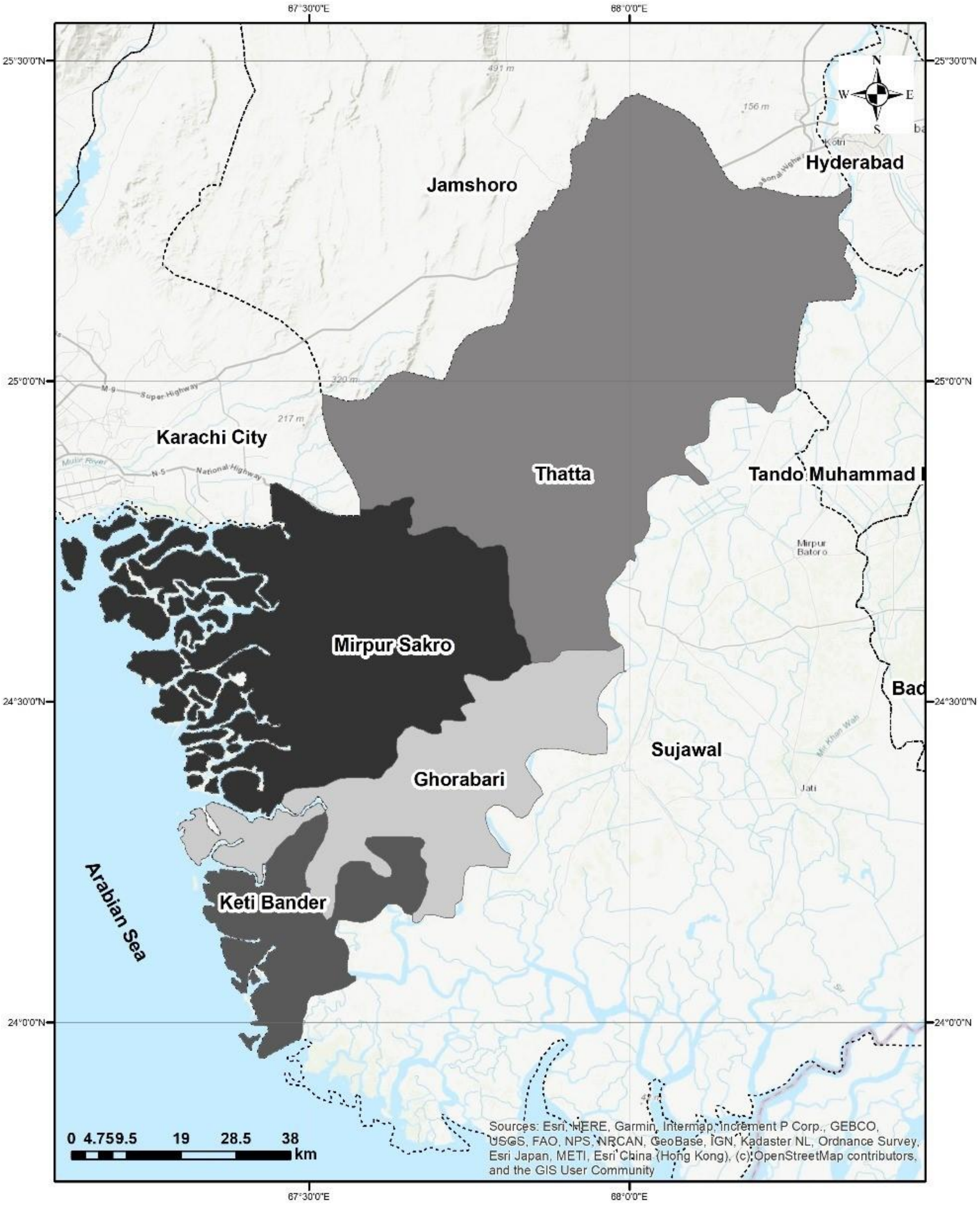

Figure 1 District and sub-districts/Talukas boundaries of Thatta

Figure 2 Rural and urban prevalence of self-reported illnesses in sub-districts of Thatta

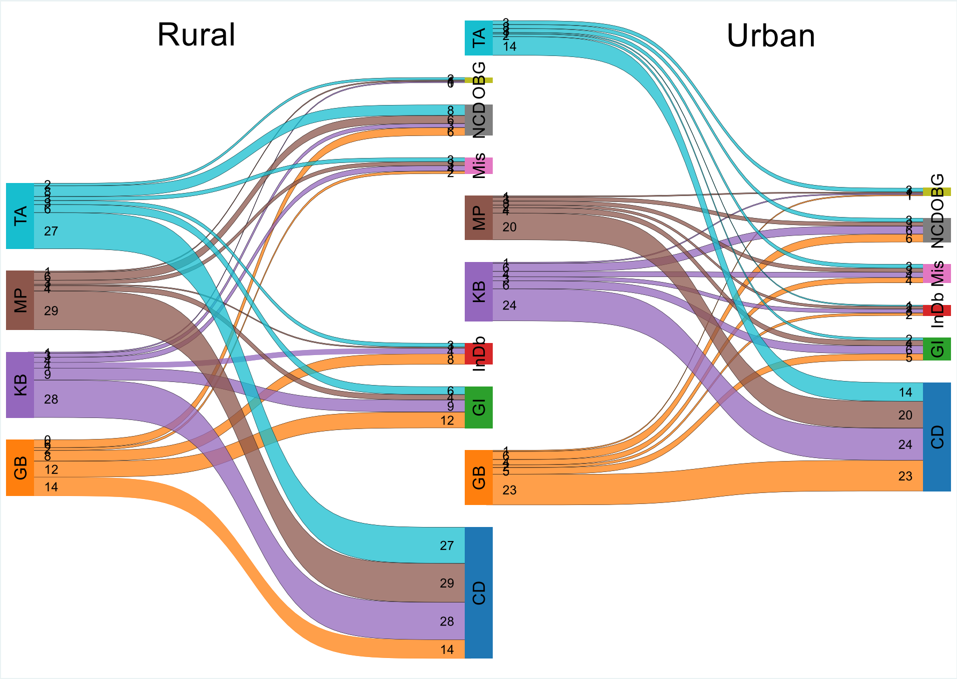

Subdistricts GB: Ghorabari, KB: Keti Bunder, MP: Mirpur Sakro, TA: Thatta
CD: Communicable Disease, NCD: Non Communicable Diseases Mental Health Disorders, GI: Gastrointestinal & Liver Disorders
InDb:Injuries & Disabilities, OBG: Gynaecology & obstetrics, Mis: Other disorders
