# Supplementary material for: Self-reported illnesses in Thatta: Evidence from a rural and underdeveloped district in Sindh province, Pakistan
Source: PLoS One. 2025 Jan 31;20(1):e0293790. doi: 10.1371/journal.pone.0293790 (PMC11785293; doi:10.1371/journal.pone.0293790)
Supplement: Attachment — Submitted filename: ReviewreportPLOSone23.docx [file pone.0293790.s002.docx]

Journal requirements:

When submitting your revision, we need you to address these additional requirements.

1. Please ensure that your manuscript meets PLOS ONE's style requirements, including those for file naming. The PLOS ONE style templates can be found at

https://journals.plos.org/plosone/s/file?id=wjVg/PLOSOne_formatting_sample_main_body.pdf and

https://journals.plos.org/plosone/s/file?id=ba62/PLOSOne_formatting_sample_title_authors_affiliations.pdf

2. Please provide additional details regarding participant consent. In the ethics statement in the Methods and online submission information, please ensure that you have specified (1) whether consent was informed and (2) what type you obtained (for instance, written or verbal, and if verbal, how it was documented and witnessed). If your study included minors, state whether you obtained consent from parents or guardians. If the need for consent was waived by the ethics committee, please include this information.

If you are reporting a retrospective study of medical records or archived samples, please ensure that you have discussed whether all data were fully anonymized before you accessed them and/or whether the IRB or ethics committee waived the requirement for informed consent. If patients provided informed written consent to have data from their medical records used in research, please include this information.

We have provided details of participants consent and data collection process in details in the methods section of the revised manuscript.

3. We note that the grant information you provided in the ‘Funding Information’ and ‘Financial Disclosure’ sections do not match.

When you resubmit, please ensure that you provide the correct grant numbers for the awards you received for your study in the ‘Funding Information’ section.

The grant information is appended below and have been added in both sections

The details of WHO Registration Grant No.: 2018/824227-0, Purchase Order: 202084766, Amount: USD 50K.

4. Thank you for stating the following in the Acknowledgments Section of your manuscript:

“We gratefully acknowledge the World Health Organization, Regional Office, Eastern Mediterranean Region for providing funding for this survey. Our acknowledgments are due to the field staff of the survey for their contributions to the survey and to the officials of the district health office, Thatta for their logistic support.”

We note that you have provided funding information that is currently declared in your Funding Statement. However, funding information should not appear in the Acknowledgments section or other areas of your manuscript. We will only publish funding information present in the Funding Statement section of the online submission form.

Please remove any funding-related text from the manuscript and let us know how you would like to update your Funding Statement. Currently, your Funding Statement reads as follows:

“World Health Organization regional office of the Eastern Mediterranean Region supported this survey”

Please include your amended statements within your cover letter; we will change the online submission form on your behalf.

Reply: The acknowledgement section is edited according to the editor’s advice.

5. We note that you have indicated that data from this study are available upon request. PLOS only allows data to be available upon request if there are legal or ethical restrictions on sharing data publicly. For more information on unacceptable data access restrictions, please see http://journals.plos.org/plosone/s/data-availability#loc-unacceptable-data-access-restrictions.

In your revised cover letter, please address the following prompts:

a) If there are ethical or legal restrictions on sharing a de-identified data set, please explain them in detail (e.g., data contain potentially sensitive information, data are owned by a third-party organization, etc.) and who has imposed them (e.g., an ethics committee). Please also provide contact information for a data access committee, ethics committee, or other institutional body to which data requests may be sent.

b) If there are no restrictions, please upload the minimal anonymized data set necessary to replicate your study findings as either Supporting Information files or to a stable, public repository and provide us with the relevant URLs, DOIs, or accession numbers. For a list of acceptable repositories, please see http://journals.plos.org/plosone/s/data-availability#loc-recommended-repositories.

We will update your Data Availability statement on your behalf to reflect the information you provide.

Reply: We cannot share data publicly because of legal and ethical restrictions. We have committed with the respondents of the survey at the time of singing consent form on confidentiality of personal information that are not to be shared with any third party. Same commitment has been made in the ethical review application." However, we can consider individual request for accessing data by writing to [zaheer.habib@aku.edu](mailto:zaheer.habib@aku.edu) Senior Manager, Data Management Unit, Community Health Sciences Department, Aga Khan University.

6. Please amend your manuscript to include your abstract after the title page.

Reply: Abstract is appended after the title page.

7. Please include a separate caption for each figure in your manuscript.

8. We note that Figure 1 in your submission contains [map/satellite] images which may be copyrighted. All PLOS content is published under the Creative Commons Attribution License (CC BY 4.0), which means that the manuscript, images, and Supporting Information files will be freely available online, and any third party is permitted to access, download, copy, distribute, and use these materials in any way, even commercially, with proper attribution. For these reasons, we cannot publish previously copyrighted maps or satellite images created using proprietary data, such as Google software (Google Maps, Street View, and Earth). For more information, see our copyright guidelines: http://journals.plos.org/plosone/s/licenses-and-copyright.

We require you to either (1) present written permission from the copyright holder to publish these figures specifically under the CC BY 4.0 license, or (2) remove the figures from your submission:

a. You may seek permission from the original copyright holder of Figure 1 to publish the content specifically under the CC BY 4.0 license.

We recommend that you contact the original copyright holder with the Content Permission Form (http://journals.plos.org/plosone/s/file?id=7c09/content-permission-form.pdf) and the following text:

“I request permission for the open-access journal PLOS ONE to publish XXX under the Creative Commons Attribution License (CCAL) CC BY 4.0 (http://creativecommons.org/licenses/by/4.0/). Please be aware that this license allows unrestricted use and distribution, even commercially, by third parties. Please reply and provide explicit written permission to publish XXX under a CC BY license and complete the attached form.”

Please upload the completed Content Permission Form or other proof of granted permissions as an "Other" file with your submission.

In the figure caption of the copyrighted figure, please include the following text: “Reprinted from [ref] under a CC BY license, with permission from [name of publisher], original copyright [original copyright year].”

b. If you are unable to obtain permission from the original copyright holder to publish these figures under the CC BY 4.0 license or if the copyright holder’s requirements are incompatible with the CC BY 4.0 license, please either i) remove the figure or ii) supply a replacement figure that complies with the CC BY 4.0 license. Please check copyright information on all replacement figures and update the figure caption with source information. If applicable, please specify in the figure caption text when a figure is similar but not identical to the original image and is therefore for illustrative purposes only.

The following resources for replacing copyrighted map figures may be helpful:

USGS National Map Viewer (public domain): http://viewer.nationalmap.gov/viewer/

The Gateway to Astronaut Photography of Earth (public domain): http://eol.jsc.nasa.gov/sseop/clickmap/

Maps at the CIA (public domain): https://www.cia.gov/library/publications/the-world-factbook/index.html and https://www.cia.gov/library/publications/cia-maps-publications/index.html

NASA Earth Observatory (public domain): http://earthobservatory.nasa.gov/

Landsat: http://landsat.visibleearth.nasa.gov/

USGS EROS (Earth Resources Observatory and Science (EROS) Center) (public domain): http://eros.usgs.gov/#

Natural Earth (public domain): <http://www.naturalearthdata.com/>

We have removed the Figure 1 map of Thatta district due to copy right issues raised above

9. Please include your tables as part of your main manuscript and remove the individual files. Please note that supplementary tables (should remain/ be uploaded) as separate "supporting information" files

We have included tables in the main text.

Additional Editor Comments:

This is an interesting study which has potential to inform healthcare utilization. However, as pointed out by reviewers, major and substantial revisions are needed before it can be accepted for publication. In addition to specific comments, including those in the manuscripts by Reviewer 2, the authors may consider the following:

While the authors suggest the use of SRI to direct priorities in health systems, essentially an exercise in burden of disease estimation, the data presented does not support this assertion. The term "burden of disease" is repeatedly mentioned. Instead, it would be more accurate to use this data to understand what communities are feeling in terms of symptoms/ disease self perceptions and use this information to improve healthcare provision. If this was the intent, please carefully re word to communicate this.

We have removed the term burden of disease from the revised version of the manuscript and consistently used the term self-reported illnesses.

Some claims such as "our estimates of burden of disease are much higher and comprehensive than the current evidence of health seeking in Thatta" (Discussion section in abstract), need to be backed with evidence. What are the study findings being compared against. Are there BoD or healthcare seeking estimates from Thatta available. Is it the same or different in public and private sector?

We have added estimated prevalence of certain illnesses reported in national and provincial surveys to clarify our comparison.

In the introduction section, there is a need to elaborate why SRI framework was used, its applicability and limitations to the context of the study. Mention of low capacity by district adminstrators needs to be both elaborated upon and corroborated with evidence.

We have provided limitation of SRI. We have provided reference to our claims of low capacity of district administrators.

There is a need to copy edit final version before submission to avoid simple grammar and word omission errors.

We have reviewed our revised manuscript by an native English speaking scholar.

Methods:

The survey and sampling design is well described.

It must be specified how many interviews were conducted per HH, specifically, if the husband and wife were both interviewed or one interviewee per HH was selected. If latter there should be analysis of the effect of this designation on the results

It should also be highlighted that answers for children are from their parents and therefore not "self-reported".

We mentioned the process of data collection from children below 2 years of age in paragraph 3 of Survey Design and Data Collection.

Verban autopsy is mentioned in the discussion as a means to verify self-reports. The methodology used is not mentioned. There is no mention of verbal autopsy in results either.

We removed the term “verbal autopsy” and provided more details on methods of collection data from the respondents.

Line 90 mentions two methods of verification were used. Please specify #2. Were any statistical means used to measure verification?

We specified two methods of verification, during the data collection and after the data collection in the revised manuscript in the second last paragraph of section on Survey Design and Data Collection.

Assumptions of prevalence ratio methodology used may be discussed.

We provided limitations of prevalence ratios in the first paragraph of the discussion section and cited some literature in support of it. However, this is not a study on validating methods of reporting risk factors/ covariates of SRIs, but we can redo the analysis using odd ratios if the reviewer may wish.

Results:

Table 2 suggests that the prevalences are for the entire year, please confirm. The period for which self-reported illnesses were asked for is not specified.

We have provided the period of data collection in paragraph 3 of section on data collection. We also corrected the heading of table 2.

Table 2 describes prevalences. Some of these vary widely (log order differences) than national figures. For example, Hypertension of 1.24%, Diabetes: 0.73% compared with national figures of 30-50% and 17-26% respectively. There is a need to discuss these differences and their posited rationale. Diarrhea is reported at 3.27% for the year, when PSLM data suggests around 16% in 2 weeks prior to survey.

We have provided such comparisons in the discussion section and the challenges making such comparisons.

Table 4 may benefit from the use of continuous age variable. Otherwise, there is concern that some of the lower morbidity among women may be a cohort effect (women are often younger than men in surveys, whether this is true or not must be mention)

The mean age difference between males in females was 0.34 years so we don’t anticipate such bias introduced in the model. Moreover, we included >5 years and<60 years purposefully learning from literature about greater healthcare need such as Malik and Azam, 2012. (Muhammad Malik, A., & Azam Syed, S. I. (2012). Socio-economic determinants of household out-of-pocket payments on healthcare in Pakistan. *International journal for equity in health*, *11*, 1-7.)

Verbal autopsy (mentioned in discussion, line 159) is not mentioned in results.

We have removed the term verbal autopsy from the methods section and have provided more details in the section on methods of data collection.

Discussion:

There needs be a discussion of log order lower prevalence of many common conditions in this survey compared to the national samples. The assertion that prevalences are "alarmingly high" (line 154) is not corroborated by national data

We have edited this argument that comparison with national survey is not possible because of difference in age cohort focused in these surveys.

While the discussion of SRI literature (likely better placed in introduction) is extensive, it needs to clarify what is intended with this review and how this review serves this study

The objective of this review is to advocate the challenges with comparison of estimates of SRIs due to difference in survey design, period, geographical coverage, methods of analysis and data collection.

SRI data presented has not been validated and cannot be used to estimate about burden of disease

We mentioned this limitation in the revised manuscript.

Unclear what is meant with unknow SRI (lines 199-201)

We replaced “unknown” with “other” SRI

While there is extensive citing of literature in discussion, it may be supplemented by interpreting the findings AND THEN connect this interpretation to the cited literature

In Recommendations, it is suggested that a combined SRI type survey may substitute for individual disease survey. This is not justified based on the data presented. If anything, the data suggests that SRI may be massively under identify serious conditions.

In the revised manuscript, we did provide interpretation of our results from other sources in Pakistan and next we compare our findings with literature outside Pakistan.

We have updated the recommendation and conclusion section and avoided argument of SRI as a substitute to national surveys

[Note: HTML markup is below. Please do not edit.]

Reviewers' comments:

Reviewer's Responses to Questions

Comments to the Author

1. Is the manuscript technically sound, and do the data support the conclusions?

The manuscript must describe a technically sound piece of scientific research with data that supports the conclusions. Experiments must have been conducted rigorously, with appropriate controls, replication, and sample sizes. The conclusions must be drawn appropriately based on the data presented.

Reviewer #1: Partly

Reviewer #2: Partly

Reviewer #3: Yes

________________________________________

2. Has the statistical analysis been performed appropriately and rigorously?

Reviewer #1: No

Reviewer #2: Yes

Reviewer #3: Yes

________________________________________

3. Have the authors made all data underlying the findings in their manuscript fully available?

The PLOS Data policy requires authors to make all data underlying the findings described in their manuscript fully available without restriction, with rare exception (please refer to the Data Availability Statement in the manuscript PDF file). The data should be provided as part of the manuscript or its supporting information, or deposited to a public repository. For example, in addition to summary statistics, the data points behind means, medians and variance measures should be available. If there are restrictions on publicly sharing data—e.g. participant privacy or use of data from a third party—those must be specified.

The data pertains to Aga Khan University, and we are working on two additional manuscripts. Once the planned articles are published, we will deposit anonymized data into the repository of Aga Khan University and the public can request access to the data for research and teaching purpose following data sharing policy of Aga Khan University.

Reviewer #1: No

Reviewer #2: No

Reviewer #3: No

________________________________________

4. Is the manuscript presented in an intelligible fashion and written in standard English?

PLOS ONE does not copyedit accepted manuscripts, so the language in submitted articles must be clear, correct, and unambiguous. Any typographical or grammatical errors should be corrected at revision, so please note any specific errors here.

We have reviewed our revised manuscript for language by a native English-speaking person.

Reviewer #1: No

Reviewer #2: No

Reviewer #3: Yes

________________________________________

5. Review Comments to the Author

Please use the space provided to explain your answers to the questions above. You may also include additional comments for the author, including concerns about dual publication, research ethics, or publication ethics. (Please upload your review as an attachment if it exceeds 20,000 characters)

Reviewer #1: This research titled as "Self-reported illnesses in Thatta: Evidence from a rural and underdeveloped district in Sindh province, Pakistan" lacks a strong conceptual foundation. The study's premise relies on a superficial analysis of existing literature, failing to adequately highlight any discernible literature gap. An effective study typically builds upon existing research by identifying gaps or unexplored areas, contributing valuable insights to the field. However, regrettably, this manuscript appears to fall short in this regard.

The absence of a clear identification of the literature gap significantly diminishes the scholarly impact of this work. A comprehensive understanding of existing research is fundamental to conducting a meaningful study, enabling the identification of areas where new knowledge can be generated. Unfortunately, this manuscript fails to provide such a distinctive contribution to the established body of knowledge in its respective field.

For a study to be considered robust and impactful, it should not only engage with relevant literature but also delineate how it extends or challenges existing knowledge. This manuscript, regrettably, does not adequately fulfill this criterion. Hence, it may not significantly advance the field or offer novel perspectives, rendering it less impactful in contributing to the discourse within the academic realm.

In the revised manuscript we were able to identify gaps in existing evidence of disease prevalence in Pakistan. Next, we compared our finding the existing evidence from Pakistan and lastly, we explicitly provide limitation of our study.

Reviewer #2: I have completed a thorough review of your manuscript titled "Self-reported illnesses in Thatta: Evidence from a rural and underdeveloped district in Sindh province, Pakistan." I commend the effort you have invested in your research, and I recognize the importance of the manuscript's topic. However, I must convey that, upon careful examination, the manuscript requires significant revisions before it can advance further in the processing stage.

The manuscript faces challenges in terms of clarity in several sections, making it difficult for readers to seamlessly follow the content. I suggest considering a restructuring of sentences and paragraphs to enhance overall coherence.

We have thoroughly revised the manuscript to bring clarity to our research methods, finding and discussion.

Numerous grammatical errors and language issues were identified throughout the manuscript. To improve the overall quality of the writing, I recommend a thorough language editing process.

We have reviewed our revised manuscript from a native English-speaking person for language and grammar.

The references require careful attention, as some instances deviate from the journal's prescribed reference guidelines. It is essential to ensure adherence to the specified format for all references.

Reference list is updated and references style for all citations is APA.

Furthermore, a comprehensive and well-articulated conclusion is currently lacking. I encourage you to summarize your key findings and discuss their implications, providing a strong closing statement for the manuscript.

We have revised the conclusion in a comprehensive and precise fashion.

Additionally, it is important to note that I could not review the tables in their entirety, as they were not present in the submitted file. I kindly request that you ensure the inclusion of tables in your revised submission, enabling a comprehensive review of all elements of the manuscript.

We now have added the Tables and Figures in the main text.

Your attention to these revisions will significantly contribute to the overall quality and readiness of your manuscript for further processing. Please see the attached manuscript for my detailed comments.

We have provided replies on the comments made in the manuscript.

Reviewer #3: It would be good to review data tables for easier understanding of key findings. Tables were not available in the manuscript, however, reference to tables is given. Maybe there is an error in uploading / downloading.

We now have added the Tables and Figures in the main text.

Tone of some sentences may be corrected, e.g. rather than "illiterate" it would be better to use the word un-educated.

Instead of years of schooling, categorization of the question on literacy were “read and write” related that is why we used literate.

Since the study focuses on just one district, it should be mentioned as a limitation of the study.

We mentioned this limitation in paragraph 6 of the discussion section.

________________________________________

6. PLOS authors have the option to publish the peer review history of their article (what does this mean?). If published, this will include your full peer review and any attached files.

If you choose “no”, your identity will remain anonymous but your review may still be made public.

Do you want your identity to be public for this peer review? For information about this choice, including consent withdrawal, please see our Privacy Policy.

Reviewer #1: No

Reviewer #2: No

Reviewer #3: No

________________________________________

[NOTE: If reviewer comments were submitted as an attachment file, they will be attached to this email and accessible via the submission site. Please log into your account, locate the manuscript record, and check for the action link "View Attachments". If this link does not appear, there are no attachment files.]

While revising your submission, please upload your figure files to the Preflight Analysis and Conversion Engine (PACE) digital diagnostic tool, PACE helps ensure that figures meet PLOS requirements. To use PACE, you must first register as a user. Registration is free. Then, login and navigate to the UPLOAD tab, where you will find detailed instructions on how to use the tool. If you encounter any issues or have any questions when using PACE, please email PLOS at figures@plos.org. Please note that Supporting Information files do not need this step.

Figure 1 Map of Thatta district and Figure 2 urban and rural difference in disease prevalence are uploaded to the above mentioned web portal please.
