# Supplementary material for: Self-reported illnesses in Thatta: Evidence from a rural and underdeveloped district in Sindh province, Pakistan
Source: PLoS One. 2025 Jan 31;20(1):e0293790. doi: 10.1371/journal.pone.0293790 (PMC11785293; doi:10.1371/journal.pone.0293790)
Supplement: Attachment — Submitted filename: Rebuttal Letter to the editorPLOSone.docx [file pone.0293790.s003.docx]

Rebuttal Letter to the editor PLOSone

Dear Editor,

Thank you very much for reviewing our manuscript. We have revised the article in the light of your review. In the following paragraph, we have provided point by point reply to the suggestion you made.

1. In the Introduction section, it is unclear what is the point of this study. Is it intended as an alternative to current BoD surveys and modeling? If so a connection must be made to BoD literature, confluence points and why this study would add to the knowledge base.

*We have revised the introduction section. We have justified the need for prevalence data for all illnesses and limitations of existing disease surveys in the country.*

2. In the Introduction section, it is unclear what is the point of Para on pg 4 from line 81

*We have removed those contents and provided the rationale for conducting this study.*

3. In the Findings section, it appears that for acute/transient illnesses, only the past two weeks period is included. This would mean different things if extrapolated for an entire year or to the community as a measure of burden of SRI. Please clarify what is the denominator and if you are intending a point in time prevalence or is it for the year - and what adjustments/ analyses would be needed for each.

*We agree that we used two recalls while estimating the prevalence of illness. This strategy complies with the definition of the prevalence of disease- the number of cases of a disease at a particular time interval which does not require extrapolating to the whole year. In the discussion session, we validated our findings on the recall period used in published literature on the subject.*

4. In the Discussion section, the first sentence is unclear. What example does the study set. It should be clarified what is the value of this information and how does it enhance the knowledge in the field. Ideally one would like to see an framework where findings of this study can be linked to BoD. Also, the discussion section should open by positing what the study has shown and what salient findings would be discussed.

*We have rewritten the discussion section.* *We provide key features of our study followed by limitations in the first paragraph of the discussion section. We differentiated between a burden of disease and self-reported illnesses study.*

5. The subsequent paras in the Discussion attempt to explain the findings in the schema: SRI, then NCI, but the discussion goes back and forth with circling back to points. Better organization and flow of logic would help understand the message.

*We have grouped out discussion into first explaining the differences in the methods used in the published SRI studies and next validating out findings with published literature.*

6. Some parts of the narrative are unclear. For e.g., the sentence on Line 307 in unclear. What could not be synthesized. Prevalence with measures of variation should be available in the data collected. Is there a desire to compare these to literature. This is not clear.

7. Better overall structuring of the narrative in Intro and Discussions with attention to flow of logic would help.

*We have revamped the entire manuscript to structure the article identify the lack of evidence in priority settings and the utility of our study and identify the limitations of our study.*
